# Efficacy and safety of dutasteride with tadalafil add-on therapy in patients with lower urinary tract symptoms secondary to benign prostatic hyperplasia

**DOI:** 10.1186/s13104-022-06183-0

**Published:** 2022-09-05

**Authors:** Daisuke Gotoh, Kazumasa Torimoto, Yosuke Morizawa, Shunta Hori, Yasushi Nakai, Makito Miyake, Kiyohide Fujimoto

**Affiliations:** 1 Department of Urology, Nara Medical University, Kashihara, Japan; 2 Department of Urology, Hirao Hospital, Kashihara, Japan

**Keywords:** Dutasteride, Phosphodiesterase type 5 inhibitor, Tadalafil, Benign prostatic hyperplasia

## Abstract

**Objective:**

To evaluate the efficacy and safety of add-on therapy with the phosphodiesterase type 5 inhibitor tadalafil in Japanese men with lower urinary tract symptoms (LUTS) due to benign prostatic hyperplasia (BPH) treated with dutasteride.

**Results:**

Twenty-four patients were enrolled. The participants had a median age of 71.0 (64.8–73.0) years and a median prostate volume of 37.3 (29.7–41.8) mL as measured using transabdominal sonography. The efficacy indicators, such as International Prostate Symptom Score (IPSS), quality of life (QOL) score, night-time urinary frequency, and night-time maximum voided volume, improved significantly at 4 weeks, and the effects lasted until 24 weeks (IPSS: 9.5 vs. 17.0, QOL: 2.0 vs. 4.0, nocturia: 2.0 vs. 2.0, night-time maximum voided volume: 290.0 vs. 240.0 mL). Overactive bladder symptom score (OABSS) and sexual health inventory for men (SHIM) significantly improved at 12 weeks, and the effects lasted until 24 weeks (OABSS: 3.0 vs. 5.0, SHIM: 11.0 vs. 7.5). However, maximum urine flow and residual urine volume showed no improvement at any point. Adverse events occurred in two cases. Taken together, add-on therapy with tadalafil was effective for patients with LUTS/BPH resistant to dutasteride monotherapy. In addition, this therapy was not associated with severe adverse events.

## Introduction

The occurrence of lower urinary tract symptoms (LUTS) secondary to benign prostatic hyperplasia (BPH) and overactive bladder (OAB) is common, and their prevalence increases with age [1, 2]. Benign prostatic hyperplasia is a progressive disease found in middle-aged and older men. Clinical progression risk factors include aging, prostate enlargement, high PSA, LUTS, quality of life (QOL) disorders, and low urinary flow. Prevalence depends on the presence of symptoms and findings. According to the results of a community-based study in Japan [3, 4], if the following conditions are met—IPSS > 7 points, prostate volume > 20 mL, and maximum urine flow rate < 10 mL/s—the prevalence is 6% in the sexagenarian age range and 12% in the septuagenarian age range. According to the European Association of Urology guidelines, both α1-blockers and PDE5-Is are suggested as first-line treatments for LUTS/BPH.

The use of 5-alpha reductase inhibitors (5-ARIs) as therapeutic agents has dramatically changed the application of medical therapy for BPH; 5-ARIs inhibit 5α reductase and reduce the prostate tissue by controlling dihydrotestosterone production. Thus, 5-ARIs reduce bladder outlet obstruction, thereby improving LUTS [5, 6]. Dutasteride inhibits both isoforms of 5α reductase, type 1 and type 2. Finasteride, an unlicensed drug in Japan, inhibits only type 2. The effectiveness of finasteride is comparable to that of dutasteride in reducing prostate tissue and improving LUTS [7]. The efficacy of the 5-ARI/α1 blocker combination therapy has also been determined, and its role has been examined by two large-scale studies (MTOPS [8] and CombAT [9]). Dutasteride was first utilized in Japan in 2009, leading to a considerable decrease in the total number of BPH-related surgeries from 20,413 (2009) to 14,152 (2014) [10].

The phosphodiesterase 5 inhibitor (PDE5-I) tadalafil was approved in Japan in 2014, and previous randomized studies have demonstrated that its efficacy is similar to that of the α1-blocker tamsulosin [11, 12]. Moreover, recent meta-analysis results have suggested that PDE5-Is can substantially improve LUTS as well as erectile dysfunction (ED) in men with BPH [13]. However, reports on the efficacy and safety of tadalafil add-on therapy for BPH treatment with dutasteride are currently insufficient.

Tadalafil is often administered as an add-on or combination treatment with dutasteride. However, there are only a few published studies that investigated the effect of this combination therapy [14–17]. Wada et al. demonstrated that dutasteride add-on is a reasonable treatment alternative for male patients with LUTS who are not satisfied with tadalafil monotherapy [17]. However, there is no publication reporting the efficacy of add-on treatment with tadalafil in patients with LUTS who are not satisfied with dutasteride monotherapy.

In this study, we examined the effect of tadalafil add-on treatment in patients with LUTS responding poorly to dutasteride monotherapy. We also subjectively and objectively evaluated the consequences of long-term prescription of add-on therapy with tadalafil for dutasteride treatment-resistant BPH.

The first-line therapy for BPH includes α1-blockers. However, with this therapy, some patients experience adverse events, such as ejaculatory disorder [18]. Due to said adverse events of α1-blocker (silodosin) and dutasteride combination treatment, we decided to avoid using silodosin. Withdrawal of the α1-blocker from the combination therapy was reasonable and tolerable considering the effect of dutasteride on lower urinary tract symptoms and bladder outlet obstruction [19]. However, in this study, this effect was poor and, consequently, tadalafil was added. This study investigated the possibility that additional treatment with tadalafil might be a treatment option if dutasteride alone is ineffective in patients who discontinued the α1-blocker from the combination of the α1-blocker and dutasteride.

## Main text

### Methods

This was a retrospective observational study. A total of 24 patients admitted to the Hirao Hospital were selected from the database of the electronic medical records system between April 2017 and December 2018. Tadalafil (5 mg) was subsequently administered to the patients. The inclusion criteria were as follows: patients were over 50 years of age, diagnosed as having LUTS/BPH, and showed poor response to dutasteride monotherapy for ≥ 6 months (International Prostate Symptom Score [IPSS] of ≥ 8 and/or IPSS-QOL index of ≥ 3). The exclusion criteria were as follows: patients with insufficient clinical data, additional use of β_3_-adrenoceptor agonist or anticholinergic drug within 3 months prior to the day of initial evaluation, a history of urinary retention or prostatic surgery, neurologic bladder, bladder stones, or a history or evidence of prostate cancer. For patients with a serum prostate-specific antigen concentration > 4 ng/mL, we performed appropriate magnetic resonance imaging and prostate biopsies as needed to rule out prostate cancer.

**Fig. 1:**
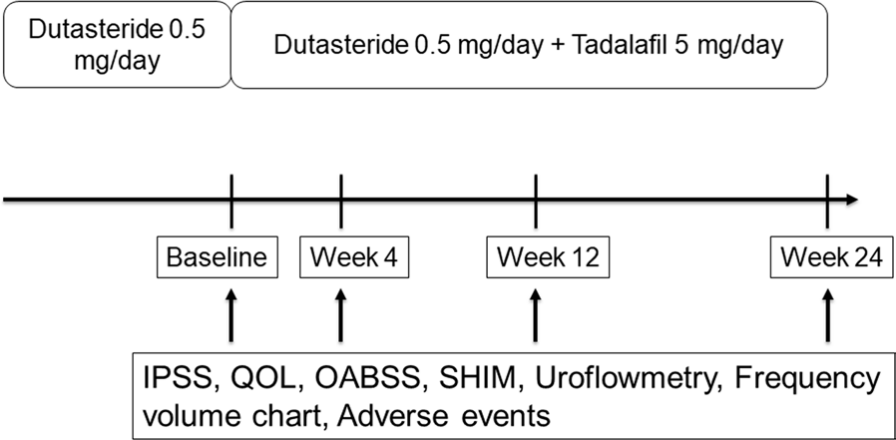
Illustration of the study protocol. The efficacy measures included IPSS, QOL score, OABSS, SHIM, UFM, and FVC at baseline and 4, 12, and 24 weeks after administration of tadalafil. Safety was evaluated based on the patient-reported adverse events during visits. IPSS, International Prostate Symptom Score; QOL, Quality of life; OABSS, overactive bladder symptom score; SHIM, sexual health inventory for men; UFM, uroflowmetry; FVC, frequency volume chart

The efficacy measures included IPSS, QOL score, overactive bladder symptom score (OABSS), sexual health inventory for men (SHIM), maximum urine flow (Qmax), post-void residual urine volume, and frequency volume chart (FVC) at baseline and at 4, 12, and 24 weeks after the administration of tadalafil (Fig. 1). Safety was evaluated based on subject-reported adverse events during visits.

IPSS score refers to the total IPSS and IPSS subscores, including Q1, incomplete emptying; Q2, daytime frequency; Q3, intermittency; Q4, urgency; Q5, weak stream; Q6, straining; Q7, nocturia; voiding symptom subscore (Q1 + Q3 + Q5 + Q6); storage symptom subscore (Q2 + Q4 + Q7); and QOL index. The OABSS is a four-item questionnaire that collectively expresses OAB symptoms in terms of a single score. The OABSS was initially developed and validated psychometrically in Japan. For FVC, we evaluated the nocturnal polyuria index and night-time maximum voided volume.

#### Statistical analysis

All values are expressed as the mean ± standard deviation. The Wilcoxon matched-pairs test was used to evaluate statistical differences. Prism software ver. 8.4.2 (GraphPad Software, San Diego, CA, USA) was used for statistical analyses and data plotting. Statistical significance was set at *P* < 0.05.

### Results

#### Participants

Twenty-four participants were enrolled in this study. Overall, the mean patient age was 71.0 (64.8–73.3) years, and the mean prostate volume examined by transabdominal sonography was 37.3 (29.9–42.9) mL (Table 1). Regarding comorbidities, 1 case of hypertension and 2 cases of diabetes were observed.

**Table 1:** Patient characteristics

	Patients (n = 24)
Age (years)	71.0 (64.8–73.3)
Prostate volume (mL)	37.3 (29.9–42.9)
PSA level (ng/mL)	1.7 (0.8–3.1)
Total IPSS	17.5 (14.0–21.0)
QOL score	4.0 (3.0–5.0)
Total OABSS	5.0 (3.0–6.0)
SHIM	7.5 (2.8–12.0)
Night-time frequency (number)	2.0 (2.0–3.0)
NPi	0.4 (0.3–0.4)
Night-time maximum voided volume (mL)	240.0 (165.0–257.5)
Maximum flow rate (mL/s)	9.7 (7.4–13.7)
Residual urine volume (mL)	52.5 (25.0–87.8)

Data are presented as the median (interquartile range)

PSA, prostate-specific antigen; IPSS, international prostate symptom score; QOL, quality of life; OABSS, overactive bladder symptom score; SHIM, sexual health inventory for men; NPi, Nocturnal polyuria index

#### Efficacy

The total IPSS was significantly lower at 4 weeks than at baseline (13.0 [10.3–16] vs. 17.0 [14.0–21.0], *P* = 0.0002) and continued to decrease for 24 weeks (9.5 [8.8–13.3] vs. 17.0 [14.0–21.0], *P* = 0.0004). The voiding symptom subscore was significantly lower at 4 weeks than at baseline (8.0 [4.0–10.0] vs. 9.0 [8.0–12.0], *P* = 0.0118) and continued to decrease for 24 weeks (5.5 [4.8–7.5] vs. 9.0 [8.0–12.0], *P* = 0.0009). Storage symptom subscore was significantly lower at 4 weeks than at baseline (5.0 [4.0–8.8] vs. 7.0 [6.0–9.0], *P* = 0.0008), and continued to decrease for 24 weeks (4.0 [3.0–5.3] vs. 7.0 [6.0–9.0], *P* = 0.0011). The QOL score was significantly lower at 4 weeks than at baseline (3.0 [2.0–3.8] vs. 4.0 [3.0–5.0], *P* = 0.0014) and continued to decrease for 24 weeks (2.0 [2.0–2.5] vs. 4.0 [3.0–5.0], *P* = 0.0021). Total OABSS was significantly lower at 12 weeks than at baseline (4.0 [2.3–6.0] vs. 5.0 [3.0–6.0], *P* = 0.0480) and continued to decrease for 24 weeks (3.0 [3.0–4.0] vs. 5.0 [3.0–6.0], *P* = 0.0248). SHIM was significantly higher at 12 weeks than at baseline (10.5 [5.0–16.3] vs. 7.5 [2.3–12.0], *P* = 0.0305) and continued to decrease for 24 weeks (11.0 [4.5–16.5] vs. 7.5 [2.3–12.0], *P* = 0.0187). Night-time urinary frequency (nocturia) was significantly lower at 4 weeks than at baseline (2.0 [1.0–2.0] vs. 2.0 [2.0–3.0], *P* = 0.0031) and continued to decrease for 24 weeks (2.0 [1.0–2.0] vs. 2.0 [2.0–3.0], *P* = 0.0103). Night-time maximum voided volume was significantly higher at 4 weeks than at baseline (270.0 [230.0–300.0] vs. 240.0 [157.5–275.0] mL, *P* = 0.0385) and continued to increase for 24 weeks (290.0 [240.0–310.0] vs. 240.0 [157.5–275.0] mL, *P* = 0.0248). However, nocturnal polyuria index, maximum urine flow rate, and residual urine volume showed no improvement at any point (Table 2).

**Table 2:** Comparison of subjective and objective parameters at baseline vs. at 4, 12, and 24 weeks

	Baseline	Week 4	Week 12	Week 24
Total IPSS	17.0 (14.0–21.0)	13.0 (10.3–16.0)***	13.5 (8.8–16.0)***	9.5 (8.8–13.3)***
Q1. Incomplete emptying	2.0 (1.0–3.0)	2.0 (1.0–2.0)*	1.0 (1.0–3.0)	1.0 (0.8–2.3)*
Q2. Daytime frequency	3 (2.3–3.0)	3.0 (2.0–3.0)**	2.0 (2.0–3.0)**	1.0 (1.0–2.0)***
Q3. Intermittency	2.5 (2.0–3.0)	2.0 (1.0–3.0)	1.0 (1.0–3.0)**	1.0 (1.0–2.0)**
Q4. Urgency	2.0 (1.0–3.0)	1.0 (1.0–2.0)**	2.0 (1.0–2.0)*	1.0 (1.0–2.0)*
Q5. Weak stream	3.0 (2.0–4.0)	2.5 (2.0–3.0)**	2.0 (1.8–3.0)***	2.0 (2.0–2.3)**
Q6. Straining	2.0 (1.0–3.0)	1.0 (1.0–2.0)	1.0 (1.0–2.0)*	1.0 (1.0–1.0)*
Q7. Nocturia	2.0 (2.0–3.0)	2.0 (1.0–2.0)**	2.0 (1.0–2.3)**	2.0 (1.0–2.0)*
Voiding symptom subscore	9.0 (8.0–12.0)	8.0 (4.0–10.0)*	7.0 (5.0–9.3)***	5.5 (4.8–7.5)***
Storage symptom subscore	7.0 (6.0–9.0)	5.0 (4.0–8.8)***	5.5 (4.0–7.0)**	4.0 (3.0–5.3)**
QOL score	4.0 (3.0–5.0)	3.0 (2.0–3.8)**	3.0 (2.0–3.0)***	2.0 (2.0–2.5)**
Total OABSS	5.0 (3.0–6.0)	4.5 (3.0–6.0)	4.0 (2.3–6.0)*	3.0 (3.0–4.0)*
SHIM	7.5 (2.3–12.0)	10.5 (4.3–13.5)	10.5 (5.0–16.3)*	11.0 (4.5–16.5)*
NPi	0.4 (0.3–0.5)	0.4 (0.4–0.4)	0.4 (0.3–0.4)	0.3 (0.3–0.4)
Night-time maximum voided volume (mL)	240.0 (157.5–275.0)	270.0 (230.0–300.0)*	300.0 (250.0–340.0)*	290.0 (240.0–310.0)*
Maximum flow rate (mL/s)	9.4 (7.3–13.8)	10.0 (6.3–13.9)	10.6 (5.8–14.0)	11.0 (6.4–12.7)
Residual urine volume (mL)	47.0 (21.0–81.5)	31.5 (17.0–76.8)	44.0 (20.0–93.5)	38.0 (0.0–76.8)

Data are presented as the median (interquartile range)

IPSS, international prostate symptom score; voiding symptom subscore (Q1 + Q3 + Q5 + Q6); storage symptom subscore (Q2 + Q4 + Q7); QOL, quality of life; OABSS, overactive bladder symptom score; SHIM, sexual health inventory for men; NPi, nocturnal polyuria index

^*^P < 0.05; **P < 0.01; ***P < 0.001, compared with baseline

#### Safety

Adverse events occurred in two cases. Rash and muscle pain were observed within 2 weeks of tadalafil administration and were classified as grade 1 (Common Terminology Criteria for Adverse Events). However, symptoms improved with drug suspension.

### Discussion

This study demonstrated that add-on therapy with tadalafil might be effective for patients with LUTS/BPH resistant to dutasteride monotherapy. This study is vital because add-on therapy with tadalafil for LUTS/BPH treated with dutasteride monotherapy showed improvement in LUTS and sexual dysfunction after 24 weeks. Several studies have reported a combination therapy using PDE5-Is and 5-ARIs for LUTS [20, 21]; however, few studies also suggest using PDE5-I as an add-on therapy secondary to 5-ARI for LUTS.

Various medicinal options are now available for BPH treatment. However, any therapeutic method or medicine for BPH may lead to sexual dysfunction, such as ED or ejaculatory disorder, in patients due to adverse effects. α1-Blockers have been used extensively for BPH therapy for a long time. Adverse events with α1-blockers mainly include orthostatic hypotension, but ejaculatory disorders are also often detected [16]. Ejaculatory disorder at increased frequency is observed with silodosin, tamsulosin, and naftopidil treatment (24.4%, 16.7%, and 7.4%, respectively) [22, 23]. In addition, tamsulosin and dutasteride combination therapy has been reported to be associated with a decrease in sexual satisfaction [24].

In the evaluation of urodynamic studies, it has been reported that dutasteride improves LUTS even after 1 year [6]. However, sexual dysfunction can be caused by 5-ARIs. It is reported that erectile function should be assessed, and ED due to dutasteride should be treated in those whose erectile function is relatively maintained, especially in young individuals [25].

BPH/LUTS is an age-independent predictor of ED, and the severity of BPH/LUTS is associated with the intensity of ED [26]. Since the underlying mechanism is common to both BPH/LUTS and ED, hyperactivity of the sympathetic system, ischemia of the vascular bed in the pelvis, a decrease in nitric oxide, and the possibility of Rho-kinase activation have been suggested [27]. It is believed that ED is caused by 5-ARIs, including both finasteride and dutasteride. The frequency of ED was 1.7–11% with finasteride and 3.4–15.8% with dutasteride in an RCT, compared with those in the placebo [28]. Dutasteride has often been used during erectile insufficiency and in patients with prostate volume > 30 mL along with α1-blockers or PDE5-Is alone. The possibility of treating sexual dysfunction using dutasteride combined with PDE5-Is has been suggested [21].

In the present study, tadalafil was used as add-on therapy in dutasteride treatment-resistant BPH patients. Dutasteride was used in combination with an α1-blocker (silodosin) in all cases; however, silodosin was discontinued since it caused an ejaculatory disorder. Regarding sexual function, the SHIM score before the tadalafil combination was 7.5, owing to which, many severe cases were reported. This is because, as anticipated, most cases were elderly patients receiving dutasteride therapy. Improvement of subjective symptoms, such as LUTS and SHIM, was detected in patients. Tadalafil was reported to control a decrease in erectile function caused by dutasteride and possibly also improve LUTS. However, changes in objective findings, such as uroflowmetry, were not detected. It was anticipated that improvement in QOL and sexual dysfunction led to subjective improvement. Moreover, the night-time urinary frequency decreased. This is due to an increase in night-time urine volume. Furthermore, storage symptoms improved in patients receiving tadalafil add-on therapy.

## Conclusions

Tadalafil add-on treatment for BPH patients during dutasteride treatment was effective in treating LUTS and sexual dysfunction and did not cause severe adverse events, suggesting that it can be safely used for combination therapy.

## Limitations

This study has some limitations. First, this was a retrospective, non-randomized study without placebo control. Second, our sample size was too small to draw a definitive conclusion. Studies with a significant number of patients are required to confirm our findings. Third, we did not perform a urodynamic study in almost all patients. It is necessary to measure the change in the bladder outlet obstruction index and voiding detrusor pressure at maximum flow rate in future studies.

## Data Availability

The datasets used and/or analyzed during the current study are available from the corresponding author on reasonable request.

## Abbreviations

IPSS: International Prostate Symptom Score
QOL: Quality of life
OABSS: Overactive bladder symptom score
SHIM: Sexual health inventory for men
FVC: Frequency volume chart
LUTS: Lower urinary tract symptoms
BPH: Benign prostatic hyperplasia
OAB: Overactive bladder
ED: Erectile dysfunction
